# Wrinkled TiNAgNW Nanocomposites for High-Performance Flexible Electrodes on TEMPO-Oxidized Nanocellulose

**DOI:** 10.3390/nano14141178

**Published:** 2024-07-10

**Authors:** Loïk Gence, Franck Quero, Miguel Escalona, Robert Wheatley, Birger Seifert, Donovan Diaz-Droguett, María José Retamal, Sascha Wallentowitz, Ulrich Georg Volkmann, Heman Bhuyan

**Affiliations:** 1 Functional Materials & Devices Laboratory, Pontificia Universidad Católica de Chile, Santiago 7820436, Chile; 2 Instituto de Física, Pontificia Universidad Católica de Chile, Avenida Vicuña Mackenna 4860, Santiago 7820436, Chilevolkmann@uc.cl (U.G.V.); hbhuyan@uc.cl (H.B.); 3 Centro de Investigación en Nanotecnología y Materiales Avanzados (CIEN-UC), Av. Vicuña Mackenna 4860, Santiago 7820436, Chile; 4 Laboratorio de Nanocelulosa y Biomateriales, Departamento de Ingeniería Química, Biotecnología y Materiales, Facultad de Ciencias Físicas y Matemáticas, Universidad de Chile, Avenida Beauchef 851, Santiago 8370459, Chile; fquero@ing.uchile.cl; 5 Millennium Science Initiative Program—Millennium Institute for Research in Optics (MIRO), Santiago, Chile; 6 Centro de Energía UC, Av. Vicuña Mackenna 4860, Macul, Santiago 7820436, Chile; 7 Facultad de Ingeniería, Universidad Finis Terrae, Santiago 7501015, Chile

**Keywords:** nanocellulose, nanocomposite, nanowires, bendable, titanium nitride

## Abstract

In this study, we present a novel method for fabricating semi-transparent electrodes by combining silver nanowires (AgNW) with titanium nitride (TiN) layers, resulting in conductive nanocomposite coatings with exceptional electromechanical properties. These nanocomposites were deposited on cellulose nanopaper (CNP) using a plasma-enhanced pulsed laser deposition (PE-PLD) technique at low temperatures (below 200 °C). Repetitive bending tests demonstrate that incorporating AgNW into TiN coatings significantly enhances the microstructure, increasing the electrode’s electromechanical robustness by up to four orders of magnitude compared to commercial PET/ITO substrates. Furthermore, the optical and electrical conductivities can be optimized by adjusting the AgNW network density and TiN synthesis temperature. Our results also indicate that the nanocomposite electrodes exhibit improved stability in air and superior adhesion compared to bare AgNW coatings.

## 1. Introduction

Within the last decade, cellulose nanofiber-based substrates, also called cellulose nanopapers (CNP), have gained a lot of attention owing to their remarkable properties, including their mechanical strength, transparency, low thermal expansion coefficient, bio-compatibility, and bio-degradability [1,2,3,4]. This low-cost and renewable material, used as a substrate or matrix, is of great interest for a multitude of emerging applications like solar cells, lithium-based batteries [5,6], and artificial electronic skin [7,8].

The development of future flexible electronics requires innovative solutions for lighter energy storage devices, as well as electrodes and interconnections that are key components for all flexible electronics devices [9]. Any improvement of their electromechanical robustness is highly desirable to limit the failure of flexible electronics. Ideal flexible electrode coatings should present a high electrical conductivity that is independent of the mechanical strain and exhibit an excellent adhesion to the substrate for avoiding delamination. Silver nanowire (AgNW) networks are good candidates for flexible transparent conductive electrodes (TCE); however, they exhibit high roughness, low adhesion to substrates, and are too sensitive to atmospheric corrosion [10]. Several methods have been reported to improve AgNW adhesion and roughness, using mechanical or temperature treatments [11,12]. Other works have suggested that wrinkled coatings offer higher mechanical strength than equivalent planar coatings [13,14].

Alongside the mechanical resistance, significant efforts have been made to develop novel materials that are also bio-compatible for integration into wearable devices. Such a material that would also possess antimicrobial or antifouling [15] properties for preventing microbial growth onto devices’ surfaces, which would enable its use for future implantable medical devices. Many strategies have been reported for designing germicide surfaces, and these can be classified as a function of the antimicrobial mechanisms. The hydrophobicity and topography of surfaces can be employed for controlling the initial adhesion, adsorption, or accumulation of the first bacterial colonizers and therefore biofouling succession, while a biocidal reagent dispersed in a matrix to form the coating layer can effectively kill the approaching microbes and prevent surface contamination [16]. Nanostructures of various metals, like copper, silver, or tin, are known to exhibit antimicrobial activity [17,18,19]. While tin-based biocides are toxic to the marine environment and human beings, silver nanoparticles have been incorporated, for more than a decade, in a multitude of medical devices and commercial products [20]. These nanostructures are generally incorporated in a matrix to form a nanocomposite used as a coating layer, deposited on the substrate. Titanium nitride (TiN) is a ceramic material that combines attractive properties such as low electrical resistivity, high melting point, and hardness with an excellent resistance to corrosion [21]. Moreover, TiN exhibits low cytotoxicity [22] and has been used for decades as a wear-reducing, allergy-resistant, and biocompatible layer for orthopedic implants [23]. A wide range of techniques are available for the synthesis of TiN coatings; these include RF or DC sputtering [24,25,26], pulsed laser deposition (PLD) [27] metal–organic chemical vapor deposition [28], and plasma-enhanced atomic layer deposition [29]. Reactively sputtered TiN was introduced by Nicolet [30] as a diffusion barrier and has recently been used in a composite material for inverted organic LED, resulting in improved performances [31]. Generally, these high-quality TiN films, which are obtained from high-temperature processes (*T* > 500 °C) that irreversibly damage any organic materials, are obviously not compatible with flexible cellulose-based and plastic substrates. It is therefore interesting to develop innovative techniques and materials that avoid thermal damage to polymeric and organic substrates. Recently, TiN coatings with excellent uniformity and plasmonic properties comparable to gold were produced at room-temperature via sputtering and PLD, enabling the use of these techniques for deposition on polymeric substrates [32,33].

In this work, we investigate and characterize electrodes produced via low-temperature plasma-enhanced pulsed laser deposition (PE-PLD). Coatings were deposited onto nanocellulose (CNP) and PET substrates, varying the synthesis temperature (from *RT* up to 150 °C), and with thicknesses ranging from 50 to 250 nm. We believe that the combination of TiN biocompatibility with the antimicrobial and antifouling properties of silver nanostructures is attractive for bio-compatible flexible electronics. We evaluated the electromechanical robustness of these electrodes via four-wire resistance measurements in situ under cyclic bending tests and compared their performances with pure AgNW coatings and PET substrates. In the following, we show that the incorporation of AgNW into TiN coatings strongly improves the electromechanical performance of TiN, with minimal impact on their optical transparency. Moreover, the nanocomposite electrodes exhibit higher stability than pure AgNW coatings upon bending stress and ambient air exposure.

## 2. Materials and Methods

### 2.1. Plasma-Enhanced Pulsed Laser Deposition

**Figure 1:**
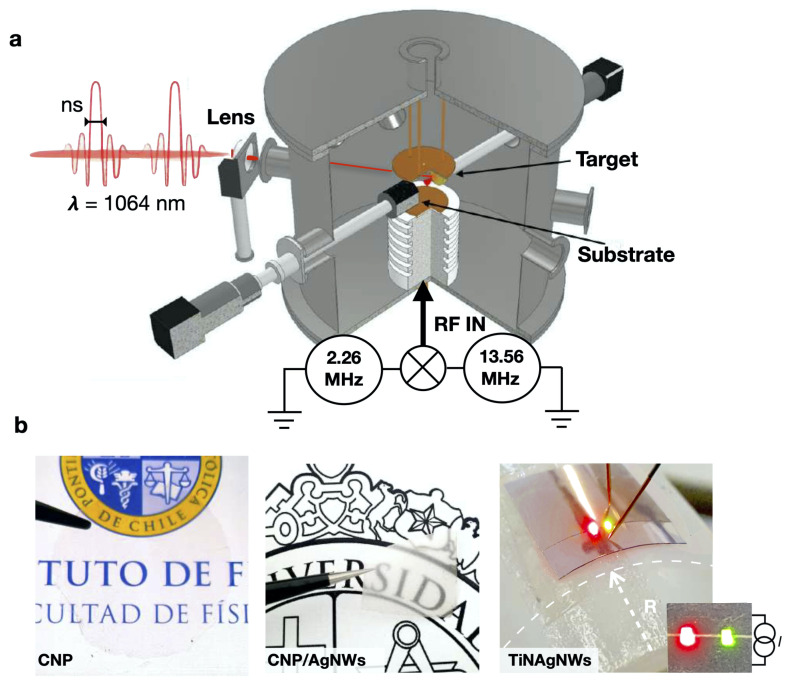
(**a**) Schematic of the plasma-enhanced pulsed laser deposition (PE- PLD) system. (**b**) Optical images of TiN- and TiNAgNW- coated nanocellulose substrates. The lower right panels show a bent TiNAgNW electrode powering two LED.

TiN coatings were deposited in a home-made plasma-enhanced pulsed laser deposition (PE-PLD) system, which combines a conventional PLD and a dual-frequency ($f_{1}$ = 13.56 MHz + $f_{2}$ = 2.26 MHz) capacitively coupled plasma (2f-CCP) system. A schematic of the system is shown in Figure 1a. The use of dual RF frequency enables the control of the nitrogen concentration and TiN deposition rate. In this work, TiN coatings on CNP and PET substrates were obtained with a constant RF power ratio ($P_{f_{1}}/P_{f_{2}}$ = 1). The average TiN deposition rate was kept constant, i.e., to approximately 10 nm·min^−1^. Further details of the experimental setup can be found elsewhere [34,35]. TiN layers were produced, varying the deposition time between 5 and 25 min, with thicknesses ranging from 50 to 250 nm, and with the synthesis temperature fixed between 25 and 150 °C.

### 2.2. Nanocomposite Electrode Fabrication

**Figure 2:**
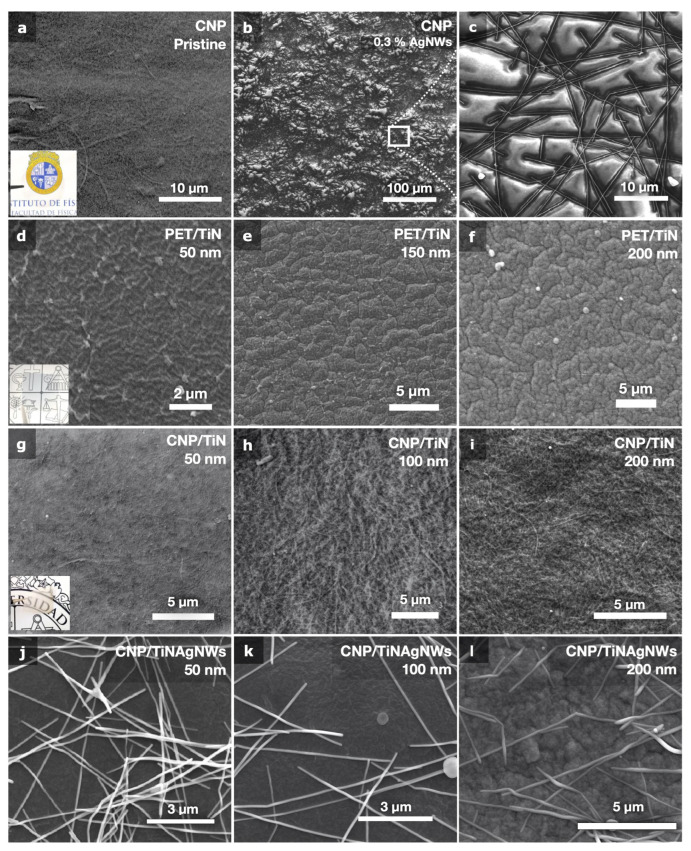
SEM images of a pristine CNP substrate (**a**), coated with silver nanowires (**b**,**c**), and TiN coatings deposited by PE-PLD at 100 °C, 20 W total RF power ($P_{f_{1}}/P_{f_{2}}$ = 1), with thicknesses between 50 an 200 nm on PET (**d**–**f**), and CNP (**g**–**i**) substrates. (**g**–**i**) The cellulose nanofibers can be distinguished for pure TiN coatings on CNP, whereas the presence of NWs modified the microstructure of the TiN layers. Wrinkles are more pronounced for thicker coatings (**f**–**l**).

Fresh CNP and PET substrates were coated with silver nanowires (AgNW) via the successive drop-casting of diluted suspensions of 70 nm diameter and 10 μm long nanowires dispersed in isopropanol (0.5%) from Sigma-Aldrich (Burlington, MA, USA). The solutions were used as received and diluted at various concentrations. Commercial biaxially oriented PET substrate, bought from Goodfellow (Huntingdon, UK), were sonicated in isopropanol and dried with nitrogen. A commercial solution of 70 and 100 nm diameter silver nanowires, 20 μm long, in isopropanol was diluted and subsequently drop-casted on 15 × 10 mm^2^ substrates for the fabrication of AgNW electrodes and the TiNAgNW nanocomposite (TiN + AgNW). The dropcasting method was optimized to increase the uniformity of the AgNW coatings (see Figure 1 and Figure 2), taking care of the drying of the sample for obtaining reproducible resistance ($\sigma_{Res}/\mu_{Res}$ < 10%). AgNW coatings were then covered by a TiN layer with thicknesses ranging from 50 up to 250 nm to form wrinkled, conducting nanocomposite coatings.

### 2.3. CNP Synthesis

The CNP substrates were produced using a commercial bleached eucalyptus cellulose pulp as the raw material. The pulp had a solid content of 13.7 wt.% and an α-cellulose content of 90.2%. Its sugar composition was 74.2% of glucans, 14.8% of xylans, and 0.8% of arabinans, among other constituents, as quantified via high-performance liquid chromatography. 2,2,6,6-Tetramethylpiperidine 1-oxyl (TEMPO) (98%), NaClO (12% solution), NaBr, and NaOH (≥99%) were purchased from Merck-Sigma Aldrich. All chemicals were used as received without further purification.

### 2.4. Characterization

**Structural analysis**. The coating microstructure of TiN coatings deposited onto a flat silicon substrate was characterized using powder X-ray diffraction (XRD) in the usual (θ−2θ) scanning configuration using a Bruker AXS D8 Advance, Cu K*α* (Bruker, Berlin, Germany), and using a step size of 0.02 degrees and a step time of 10 s. The morphology of the resulting coatings was studied via scanning electron microscopy (FE-SEM, FEI Quantum FEG 250, Hongkong, China), and EDX spectroscopy was performed at 10 KeV to identify the elemental composition of materials. Surface roughness and morphology were characterized via atomic force microscope (AFM) measurements performed with a NanoWizard3 NanoScience AFM from JPK Instruments (Berlin, Germany). Standard AFM cantilevers from Nano and More, USA, were used, employing a resonance frequency of 30 kHz and a force constant of 0.3 N/m. The 512 × 512 pixels images were recorded at a scan rate of 0.5 Hz. AFM and FE-SEM images were analyzed using Gywddion software (v 24.8).

**Electro-mechanical characterization**. The electrical resistivity was evaluated using the Van der Pauw method, with a HP Agilent 3245 Precision source (Santa Clara, CA, USA) and a high-resolution digital multimeter (DMM’s) from Keithley (Cleveland, OH, USA). Measurements were performed just after TiN deposition and, as a function of time, up to 480 days after the synthesis. To perform bending experiments, 15 mm × 10 mm coated substrates were held by two clamps, each one equipped with two thin copper electrodes. The resistance was monitored in a four-wire configuration as a function of the bending radius defined by the motion of a linear stage (2.5 μm steps).

**CNP characterization**. FTIR spectra were recorded using a Thermo Scientific Nicolet iS10 spectrometer (Waltham, MA, USA) equipped with an attenuated total reflectance accessory in the wavenumber range of 4000 to 400 cm^−1^ at a spectral resolution of 4 cm^−1^. Spectra were baseline corrected and normalized using OMINIC spectra software 9.8.286. Information about the crystalline structure of the TEMPO-CNFs was generated using a diffractometer (Bruker D8 Advance, Berlin, Germany) equipped with a monochromatic radiation source of Cu K*α* radiation (λ = 1.54 Å) at 40 kV and 30 mA. A step time of 0.02°, each 0.1 s in the diffraction angle 2θ range of 5–80°, was employed. The diffraction patterns of the samples were processed using Origin Pro Software 9.0. Thermogravimetric analysis (TGA) was conducted using a thermal analyzer (TA instruments model Q50, New Castle, DE, USA). Each sample with an initial mass of ≈10 mg was subjected to a temperature range of 20 to 800 °C at a heating rate of 10 °C min^−1^ under nitrogen atmosphere with a flow rate of 40 mL min^−1^.

## 3. Results and Discussion

### Coating Microstructure Characterization

**Figure 3:**
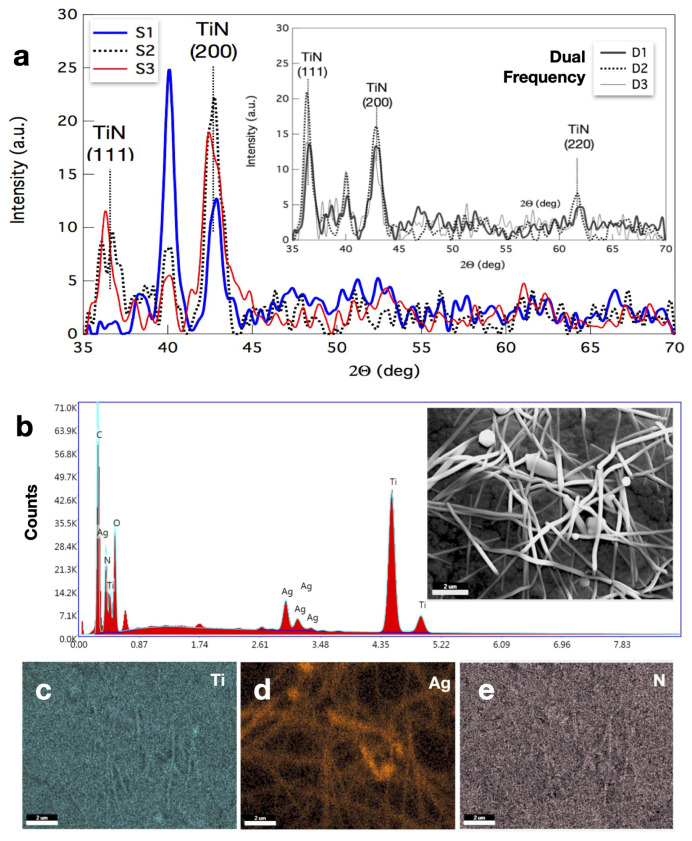
(**a**) XRD and elemental composition by EDX analysis. (**a**) XRD diffractograms of the TiN coatings produced by single- (S samples) and dual- (D samples) frequency modes. (**b**) Elemental composition of a 100 nm thick TiNAgNWs coating on CNP substrate by EDX. EDX measurement was performed at several different spots of the respective samples, and similar elemental compositions were recorded. (**c**–**e**) Alongside the presence of Ag, a uniform signal is observed for both Ti and N, suggesting an homogeneous coating composition.

We characterized the microstructure of AgNW, TiN, and TiNAgNW-based coatings deposited on CNP y PET substrates via AFM, SEM imaging, XRD, and EDX analysis. The X-ray diffraction diffractograms of TiN deposition on flat silicon substrate are shown in Figure 3a The data show that TiN coatings are multi-crystalline in the presence of background RF plasma. The TiN (111) orientation is observed for dual-frequency depositions, in contrast to the (200) orientation observed in single-frequency deposition. The increase in the ion mean energy at a similar input power in dual-frequency plasma, compared with single-frequency plasma, results in such a crystallographic orientation [35].

Figure 2 gives representative SEM micrographs of flexible PET- and CNP-coated substrates. The TiN layers were produced at 100 °C with an applied RF power of 20 W in dual-frequency mode. Despite the apparent local non-uniformity of the AgNWs dispersion shown in the SEM images of the panels (k–l) in Figure 2, a *hot dropcasting* technique enabled the production of conducting coatings with good uniformity at a larger scale (panel Figure 2b) with controlled sheet resistance values.

**Figure 4:**
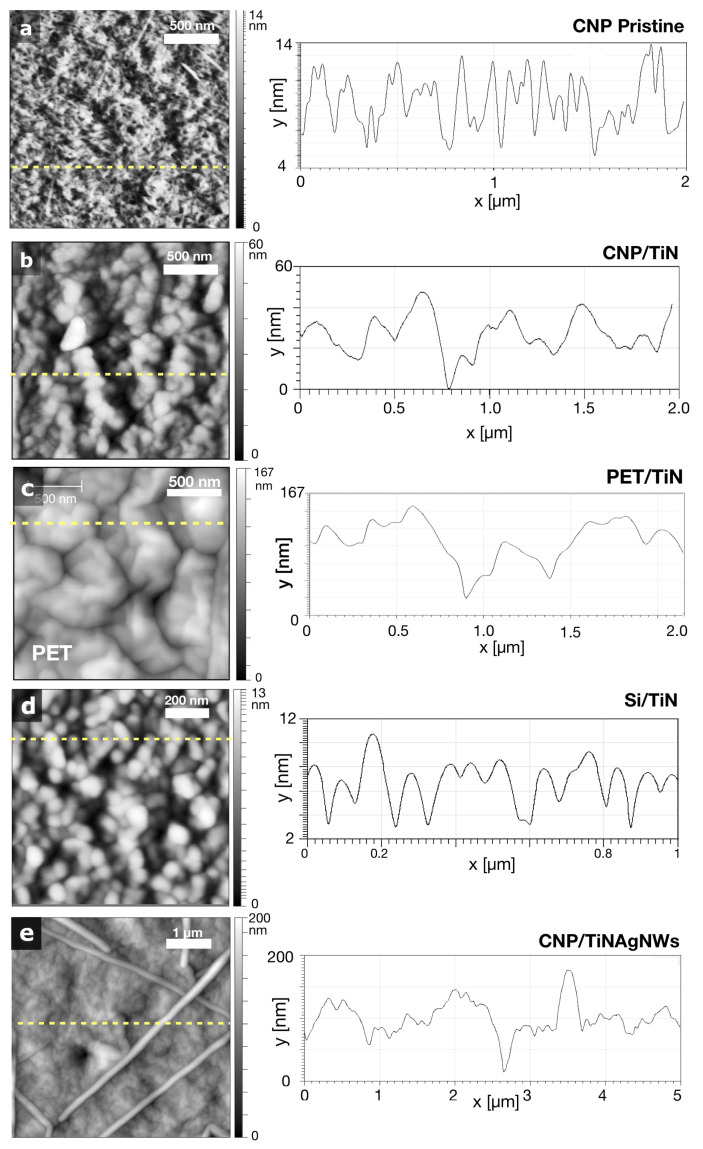
Representative AFM images of pristine CNP substrate (**a**), TiN-coated CNP (**b**) and PET (**c**) substrates. (**d**) TiN deposited on a Si/SiO_2_ substrate. (**e**) TiNAgNW coatings on CNP. TiN layers are 100 nm thick and are produced at 100 °C. The TiN grains can be distinctly seen in panels (**b**,**d**) while strong wrinkles are observed for TiNAgNW coatings on CNP.

No wrinkling or buckling is observed for TiN-coated CNP substrates, and the cellulose nanofibrils can be clearly seen, suggesting that the TiN layer is conformal to the CNP substrate topography. On the other hand, wrinkled structures are observed for TiN-coated PET substrates and strongly depend on the TiN thickness, as shown in panels Figure 2d–f. For both PET and CNP substrates, delamination phenomena or cracks were observed for synthesis temperatures above 200 °C, starting around existing defects or from the ones induced by the PLD process, and these were caused by the thermal degradation of the substrates (see Supplementary Materials). To further evaluate the morphology of these wrinkles, we performed AFM analysis. AFM images and selected profiles of pristine and coated substrates are given in Figure 4. Panel (a) shows a typical image of as-synthesized TEMPO CNP substrates. Average and rms roughnesses were obtained from 2 × 2 μm^2^ AFM images after average plane levelling of the raw data, without any further data processing. Roughness values are given in Table 1 for 100 and 250 nm thick coatings. The TiN/CNP substrates exhibit a 36 and 66% rms roughness increase compared to pristine CNP, whereas rms roughness increases by 457 and 577% for the PET substrates for 100 and 250 nm thick TiN layers, respectively. Strikingly, 100 nm thick TiN/CNP coatings (panel Figure 4b) are very similar to TiN depositions obtained on flat inorganic silicon substrates (panel Figure 4d) with comparable TiN grains size and distribution.

To explain the difference in microstructure between PET and CNP substrates, one must take into consideration the mechanical properties of both substrates and investigate wrinkling and buckling mechanisms. The bonding of thin films to substrates is a common but key process for semiconductor stacks fabrication. While the buckling mechanism generally leads to thin film failure and is not desired in standard electronic devices, the formation of wrinkles in a thin film may offer improved functionalities for flexible and stretchable electronics. Wrinkling phenomena are observed in coated flexible substrates submitted to compressive stress over a wide range of system length scales and materials [36]. The mechanical stress in coated polymeric substrates can be decomposed in two components: an intrinsic stress $\sigma_{i}$ that arises from the thin film deposition process (sputtering, spincoating, vapor deposition, *…*) and a thermal stress $\sigma_{th}$ that develops in thin films deposited above/below room temperature and that originates from the thermal expansion mismatch between the thin film and the substrate [37]:

(1) $$\sigma_{th}\propto \left(\alpha_{Sub}-\alpha_{Film}\right)\left(T_{S}-T_{RT}\right),$$

where α represents the coefficient of thermal expansion, and $T_{RT}$ and $T_{S}$ are the room and the synthesis temperature, respectively.

The interplay of these two components determines $\sigma_{T}$, the total mechanical stress applied to a coating. Wrinkling phenomena occur when $\sigma_{T}$ is positive (compressive) and exceeds a corresponding critical wrinkling stress $\sigma_{w}$ that depends on the thicknesses, Young’s modulus, and Poisson coefficient of the coating and the substrate, respectively [14,38].

TiN coatings produced at room temperature $T_{RT}$ do not exhibit such wrinkles because of the absence of thermal stress. With increasing synthesis temperature, TiN wrinkles start to form on PET substrates, and their microstructure (wavelength and amplitude) depends on the coating thickness, as shown in Figure 2d–f. The simple 1D wrinkling model [39] predicts wrinkle wavelength given by $\lambda_{0}=2\pi t_{c}{\left(\bar{E_{c}}/3\bar{E_{s}}\right)}^{1/3}$, where $\bar{E_{s}}$ and $\bar{E_{c}}$ are the film and substrate plane strain moduli, respectively [40]. We gathered, in Table 2, typical values of the CTE, Poisson ratio, and Young’s modulus of the materials constituting our samples. From these values, the theoretical wrinkle wavelengths $\lambda_{0}$ = 0.95, 1.90, and 3.82 μm are expected, respectively, for the 50, 100, and 200 nm thick TiN coatings on PET substrates. The wrinkle wavelength and its dependence on TiN thickness are in good agreement with this wrinkling model, and the wrinkle amplitude is proportional to the wrinkle wavelength, as suggested by Cerda et al. [36]. However, the observed wavelengths are 2–3 times smaller than expected for the 50 nm TiN coatings. This discrepancy can be explained by the difficulty of determining the wavelength of smaller-amplitude wrinkles.

Strikingly, the coating morphology is different for CNP substrates and strongly depends on the presence of AgNW. Images of 50 to 200 nm thick pure TiN coatings produced at 100 °C on CNP substrates are given in Figure 2g–i. No wrinkles are observed, and the RMS roughness decreases by more than 300% compared to TiN/PET. This clearly stems from the almost identical CTE values for TiN and CNP substrates (see Table 2), which results in negligible thermal stress ($\sigma_{th}\ll \sigma_{w}$) during the TiN deposition process. Nevertheless, the presence of AgNW prior to the TiN PLD deposition increases the coating roughness by a factor of 2 and introduces TiN wrinkles, which also depend on the coating thickness, as shown in Figure 2d–f.

**Figure 5:**
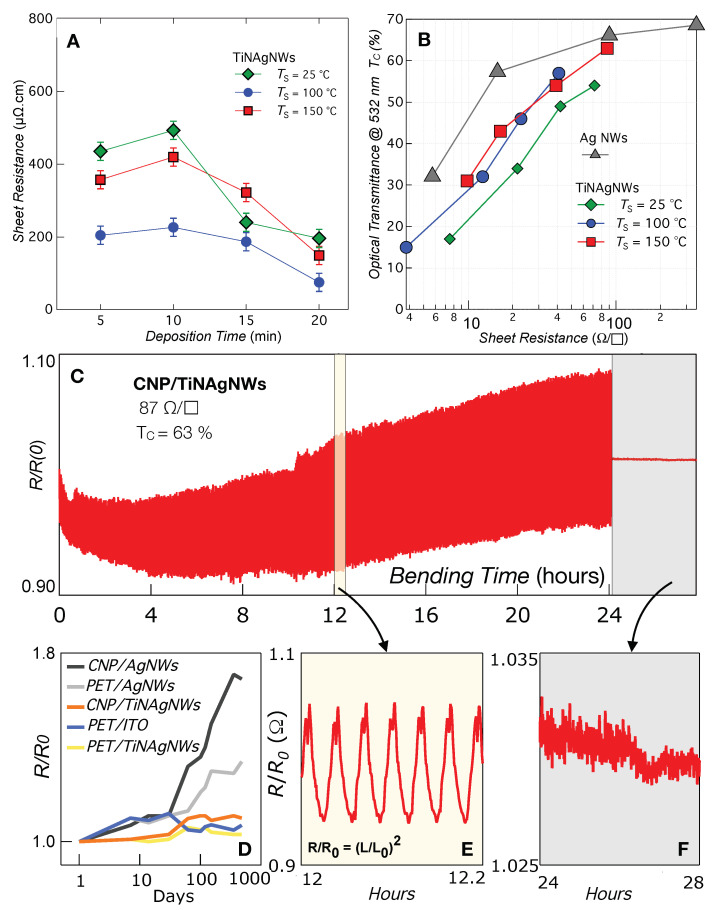
(**A**) Room temperature electrical resistivity of TiN-AgNW nanocomposites on TEMPO substrates as a function of the deposition time and synthesis temperature, using the Van der Pauw method. The samples were all prepared with the same AgNW layers of 0.3%, varying the TiN deposition parameters. (**B**) Plot of the optical transmittance as a function of the sheet resistance of the nanocomposites and pure AgNW coatings on CNP substrates. (**C**) The relative electrical resistance R/R(0) during continuous 24 h bending fatigue testing (770 cycles, $\epsilon_{max}$ = 1.5%). (**D**) the long-term stability of the conductive substrates. (**E**) Zoom of the parabolic shape of the R/R(0), suggesting that the electrical conductivity is constant during the bending cycles. (**F**) After cycling, R/R(0) exhibit excellent stability.

In the following, we describe the optoelectronic properties and mechanical robustness of these TiNAgNW coatings. As shown in Figure 5, an obvious advantage of the incorporation of AgNW in the bare TiN coatings is that it decreases the resistivity to below 100 μΩ·cm, while high-quality TiN coatings produced by high-temperature (T≥500 °C) PLD processes generally exhibit room-temperature electrical resistivity better than 300 μΩ·cm [27,50]. It is important to highlight that our AgNW coatings can be produced via dropcasting and the proper drying method with high uniformity, achieving reproducible sheet resistance (coef. of variation below 10%).

Figure 5A, shows the room temperature electrical resistivity of TiNAgNW nanocomposites as a function of the PE-PLD deposition time and processing temperature. A clear improvement of the resistivity is observed with increasing temperature and deposition time (TiN thickness). We also quantified the transparency of the produced electrodes on TEMPO substrates. The optical transmittance characterization of the produced electrodes with TEMPO substrates are shown in panel (B) of Figure 5. The optical transmittance, given at a wavelength of 532 nm, strongly depends on the TiN thickness, and a maximum of $T_{C}$ = 63% is observed for 87 Ω/□ nanocomposite coatings, as shown in Table 3. These optical transmittances are below the standard values of ITO/PET (78–80%) or pure AgNW/CNP (76%) substrates. This result probably limits the use of the mentioned nanocomposites as transparent electrodes for photovoltaic applications. However, these TiNAgNW nanocomposites exhibit enhanced mechanical properties, and surpass commercial ITO/PET substrates, in terms of electromechanical stability, by four orders of magnitude. To show that nanocomposites produced by the same PE-PLD technique are good candidates for the fabrication of flexible electrodes, we evaluated their electromechanical properties through cyclic fatigue bending tests. Samples were bent from the flat or equilibrium position to a maximum bending strain $\epsilon_{max}$. Figure 5A shows representative 24 h data of the relative resistance R/R(0) of a TiNAgNW electrode on CNP (100 nm TiN), measured in a four-probe configuration, as a function of time. During the first 4 h, the decrease in R/R(0) is attributed to an improvement in the contact resistance between the copper electrodes and the nanocomposites, and during the next cycles, the average R/R(0) value slowly increases, and only by 3.4%, after 24 h bending tests for a maximum bending stress $\epsilon_{max}$ = 1.5%; whereas an increase higher than 1000% is observed for ITO coatings on polymeric substrates [51]. We also evaluated the long-term evolution of R/R(0) for different coatings on both PET and CNP substrates exposed to air. Figure 5D gives R/R(0) as a function of the number of days of exposure to air. The data show that pure AgNW coatings are strongly impacted by air exposure with a stronger effect for CNP substrates, and they can be understood through the higher permeability to oxygen of the CNP substrate compared to PET substrates. Despite their excellent optical and electrical conductivities, AgNW coatings are characterized by high roughness, low adhesion [10] to flexible substrates, and are subject to oxidation upon air exposure. The deposition of a chemical stable layer of TiN represents an excellent opportunity to overcome the bottleneck of bare AgNW coatings for use as flexible electrodes.

## 4. Conclusions

In summary, we described and discussed the fabrication and characterization of highly conductive coatings deposited onto PET and CNP substrates using a low-temperature plasma-enhanced PLD technique. We evaluated how coating thickness, synthesis temperature, and microstructure influenced optical transmittance, electrical conductivity, and surface roughness. The coatings produced at a low temperature of T= 100 °C achieved electrical resistivity of as low as 100 μΩ while maintaining good adhesion. The electromechanical robustness of the nanocomposites was assessed through bending fatigue tests, showing that TiN-AgNW nanocomposites exhibit superior electromechanical properties and improved long-term stability compared to bare AgNW, TiN coatings, and ITO/PET substrates. Notably, these nanocomposites were more than four orders of magnitude more resistant to catastrophic failure than the commercial conductive PET alternatives. These findings suggest that conductive CNP substrates based on TiN-AgNW nanocomposites, which combine high electrical conductivity, mechanical robustness, and adjustable optical transparency, are promising materials for advancing bio-compatible flexible electronics and could lead to the development of new wearable and implantable medical devices.

## Figures and Tables

**Table 1:** Roughness extracted from AFM measurements of pristine, TiN-, and TiNAgNW-coated CNP substrates after average plane leveling of the raw data. TiN layers were deposited at 100 °C, with 20 W total RF power ($P_{f_{1}}/P_{f_{2}}$ = 1).

Samples	TiN Thickness (nm)	*R_a_* (nm)	*R_rms_* (nm)	ScanSize (μm^2^)
CNP	-	4.4	5.6	2 × 2
TiN-100/PET	100	20.5	25.6	2 × 2
TiN-100/CNP	100	5.9	7.6	2 × 2
TiN-250/PET	250	27.5	32.3	2 × 2
TiN-250/CNP	250	7.8	9.3	2 × 2
TiNAgNW-100/CNP	100	12.2	15.5	2 × 2
TiNAgNW-250/CNP	250	17.3	21.1	2 × 2

**Table 2:** Standard values for the Poisson’s ratio, coefficient of thermal expansion (CTE), and Young’s modulus of indium tin oxide (ITO), titanium nitride (TiN), polyethylene therephtalate (PET), and cellulose nanopaper (CNP).

Materials	Thickness (μm)	Poisson Ratio	CTE, *α* (10^−6^K^−1^)	Young Modulus (GPa)	References
ITO	0.100	0.15	10	1	[41,42]
TiN	0.050–0.25	0.25	6–9	400	[43,44]
PET	75–180	0.35	35	3	[37]
CNP	50–100	0.23	7	1–8	[45,46,47,48,49]

**Table 3:** Sheet resistance, optical transmittance at 550 nm, and R/R(0) increase after 1000 bending cycles with a maximum strain $\epsilon_{max}=2\%$. Samples were prepared with 0.3% AgNW solution and a variable-thickness TiN layer synthesized at $T_{S}$ = 100 °C.

Samples	*Resistance* (Ω/□)	*T_C_* (% @ 532 nm)	*R*/*R*(0) (Strain *ϵ* = 2%)
PET	-	87	-
PET/AgNW	32	78	1.27
PET/ITO	60	78	29,090
CNP/AgNW	32	76	1.22
CNP/TiN	104	49	3.93
100 nm—CNP/TiNAgNW	21	43	1.07
200 nm—CNP/TiNAgNW	13	32	1.18
50 nm—CNP/TiNAgNW	87	63	1.45

## Data Availability

Additional data that support the findings of this study are available within the article and its Supplementary Materials. The synthesis of the cellulose nanopaper (CNP) and TEMPO-oxidized CNP is presented, as well as their characterizations, including XRD, FTIR absorbance, DTG, and TGA curves.

## Supplementary Materials

The following supporting information can be downloaded at: https://www.mdpi.com/article/10.3390/nano14141178/s1, Figure S1: (a) XRD data for CNP and Tempo Samples. The peak at 2θ = 22°  indicates the presence of the crystalline region. The Inset shows a TiN-coated CNP substrate. (b) FTIR Absorbance for CNP and Tempo treated CNP samples. (c) Derivative Thermogravimetry (DTG) of CNP and CNP Tempo samples. The inset gives the corresponding TGA curves and a picture of a pristine CNP substrate; Figure S2: SEM images of TiN coatings on PET substrates produced (A) at room temperature (RT) and T = 150 °C (B). No wrinkles are observed at RT however similar wrinkles can be induced on RT TiN coating by electron-beam irradiation during FE-SEM imaging (inset). Scale bars are 2 µm. (C) picture of a fresh apple (left) and dried apple that exhibit similar skin wrinkles. Figure S3: SEM images of TiN coatings on CNP and PET substrate deposited at elevated temperature (T > 200 °C). The thermal degradation of the CNP and PET substrates further increases the compressive strain on the TiN coatings. References [52,53,54,55] are cited in the supplementary materials.

## Abbreviations

The following abbreviations are used in this manuscript:

TCE	transparent conductive electrode
PE-PLD	plasma-enhanced pulsed laser deposition
TiN	titanium nitride
CNP	cellulose nanopaper
AGNWs	silver Nanowires
PET	polyethylene terephthalate
2f-CCP	dual-frequency capacitively coupled plasma
