# Supplementary material for: Wrinkled TiNAgNW Nanocomposites for High-Performance Flexible Electrodes on TEMPO-Oxidized Nanocellulose
Source: Nanomaterials (Basel). 2024 Jul 10;14(14):1178. doi: 10.3390/nano14141178 (PMC11279476; doi:10.3390/nano14141178)
Supplement: Supplementary file 1 [file nanomaterials-14-01178-s001.zip › nanomaterials-3063432-supplementary.pdf]

nanomaterials

Article

Supplementary Information: Wrinkled TiNAgNW Nanocomposites for High- Performance Flexible Electrodes on TEMPO-Oxidized Nanocellulose

Loïk Gence [1,2,3,*], Franck Quero [4], Miguel Escalona [2], Robert Wheatley [2,5], Birger Seifert [2,3,5], Donovan Diaz-Droguett [2,3,6], María José Retamal [7], Sascha Wallentowitz [2], Ulrich Georg Volkmann [2,3] and Heman Bhuyan [2,3]

1 Functional Materials & Devices Laboratory, Pontificia Universidad Católica de Chile, Santiago 7820436, Chile
2 Instituto de Física, Pontificia Universidad Católica de Chile, Avenida Vicuña Mackenna 4860, Santiago 7820436, Chile; volkmann@uc.cl (U.G.V.); hbhuyan@uc.cl (H.B.)
3 Centro de Investigación en Nanotecnología y Materiales Avanzados (CIEN-UC), Av. Vicuña Mackenna 4860, Santiago 7820436, Chile
4 Laboratorio de Nanocelulosa y Biomateriales, Departamento de Ingeniería Química, Biotecnología y Materiales, Facultad de Ciencias Físicas y Matemáticas, Universidad de Chile, Avenida Beauchef 851, Santiago 8370459, Chile; fquero@ing.uchile.cl
5 Millennium Science Initiative Program—Millennium Institute for Research in Optics (MIRO), Santiago, Chile
6 Centro de Energía UC, Av. Vicuña Mackenna 4860, Macul, Santiago 7820436, Chile
7 Facultad de Ingeniería, Universidad Finis Terrae, Santiago 7501015, Chile
* Correspondence: logence@uc.cl

Abstract
In this study, we present a novel method for fabricating semi-transparent electrodes by combining silver nanowires (AgNW) with titanium nitride (TiN) layers, resulting in conductive nano-composite coatings with exceptional electro-mechanical properties. These nano-composites were deposited on cellulose nanopaper (CNP) using a plasma-enhanced pulsed laser deposition (PE-PLD) technique at low temperatures (below 200 °C). Repetitive bending tests demonstrate that incorporating AgNW into TiN coatings significantly enhances the microstructure, increasing the electrodes electro-mechanical robustness by up to four orders of magnitude compared to commercial PET/ITO substrates. Furthermore, the optical and electrical conductivities can be optimized by adjusting the AgNW network density and TiN synthesis temperature. Our results also indicate that the nanocomposite electrodes exhibit improved stability in air and superior adhesion compared to bare AgNW coatings.

check for updates

Citation: Gence, L.; Quero, F.; Escalona, M.; Wheatley, R.; Seifert, B.; Diaz-Droguett, D.; Retamal, M.J.; Wallentowitz, S.; Volkmann, U.G.; Bhuyan, H. Wrinkled TiNAgNW Nanocomposites for High-Performance Flexible Electrodes on TEMPO-Oxidized Nanocellulose. *Nanomaterials* **2024**, *14*, 1178. https://doi.org/10.3390/nano14141178

Academic Editors: Mohammed Jaouad Meziani and Li Cao

Received: 1 June 2024
Revised: 15 June 2024
Accepted: 19 June 2024
Published: 10 July 2024

Copyright: © 2024 by the authors. Licensee MDPI, Basel, Switzerland. This article is an open access article distributed under the terms and conditions of the Creative Commons Attribution (CC BY) license (https://creativecommons.org/licenses/by/4.0/).

CNP Fabrication

First of all, a cellulose suspension having a solid content of 0.5 wt.% was prepared by diluting the cellulose pulp having a solid content of 13.7 w%. The suspension was subsequently homogenized using a high-shear homogenizer (T-25 Digital Ultraturrax, IKA, USA) at 15 000 rpm for 2 min. The TEMPO-mediated modification of cellulose was carried out according to [1] with few modifications. The modification was carried by using adding 0.016 g of TEMPO per gram of cellulose and 0.1 g of NaBr per gram of cellulose to the previously prepared cellulose suspension. The mixture was stirred magnetically until TEMPO and NaBr were fully dissolved. Then, 0.005 mL of NaClO per gram of cellulose was added dropwise. During that step, pH was maintained to 10 by adding controlled amount of NaOH 0.5 M. The reaction occurred at room temperature and under magnetic sitting (600 rpm) until pH remained constant without further addition of NaOH, typically within 1 h. Upon completion of the reaction, the material obtained was rinsed with distilled water, vacuum filtered until neutral pH was reached. CNPs were also produced except that the cellulose used was not modified. These CNPs were used as reference material for

ATR-FTIR, powder DRX and TGA.

The solid content of the TEMPO-mediated modified cellulose was adjusted to 1 wt.%. The suspension was then processed using a kitchen blender (TH-850 D, Thomas, Germany) for 5 min at 31 000 rpm and a high-shear homogenizer (T-25 Digital Ultraturrax, IKA, USA) at 15 000 rpm for 2 min. Then the pH of the suspension was adjusted to 10 and subsequently homogenized by 1 pass through a double chamber high-pressure homogenizer (SPX, APV-2000, Denmark) at a pressure of 1000 bars. The aqueous suspension of TEMPO-cellulose nanofibers (TEMPO-CNPs) was stored in a refrigerator (4 C) until further use. The amount of carboxylic groups present at the surface of TEMPO-CNPs was quantified by conductometry and a value of 0.185 mmol/g was obtained. The TEMPO-CNPs suspension was adjusted to 0.5 wt.%. Then, the suspension was magnetically agitated a 750 rpm for 12 h. In order to improve the dispersion of TEMPO-CNPs in water, the suspension was submitted to ultrasonication for a total of 7 min at 120 W power (Sonifier 250, Branson, USA). Then, a volume of 40 mL of suspension was vacuum filtered for 3 min resulting in a wet filter cake, which was subsequently sandwiched between glass (internal) and metallic (external) plates and pressed at 20 MPa for 20 min. A film was obtained which was finally dried at 100 C for 30 min using a heat press (Rheinstern, Germany). At the end of the manufacturing process, a thin transparent/translucent film was obtained having a thickness in the range of 50–100 µm.

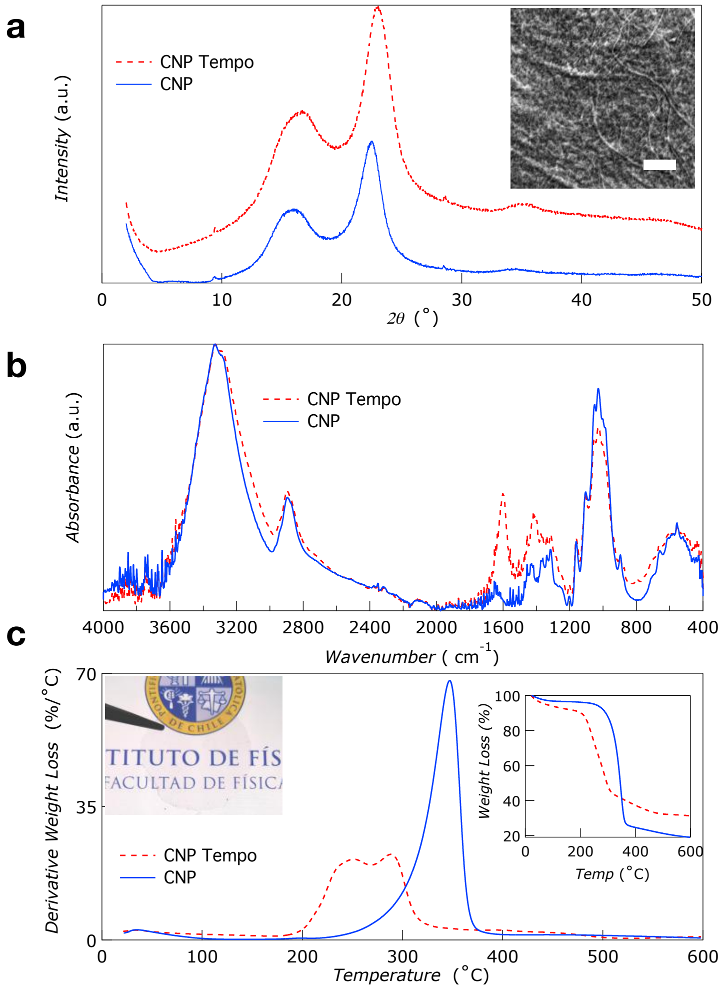

Figure S1. (a) XRD data for CNP and Tempo Samples. The peak at $2\theta = 22^o$ indicates the presence of the crystalline region. The Inset shows a TiN-coated CNP substrate. (b) FTIR Absorbance for CNP and Tempo treated CNP samples. (c) Derivative Thermogravimetry (DTG) of CNP and CNP Tempo samples. The inset gives the corresponding TGA curves and a picture of a pristine CNP substrate.

CNP Characterization

The characterization of the synthesized cellulose nanopaper (CNP) and TEMPO-oxidized CNP is presented in Figure S1. Cellulose structure can vary greatly depending on the synthesis parameters that impact the proportion of amorphous domains and its chemical stability. The pulp had a solid content of 13.7 wt. % and an α-cellulose content of 90.2%. Its sugar composition was 74.2 % of glucans, 14.8 % of xylans and 0.8 % of arabinans among other constituents as quantified by high performance liquid chromatography. 2,2,6,6-Tetramethylpiperidine 1-oxyl (TEMPO) (98 %), NaClO (12 % solution), NaBr and NaOH ($\geqslant$ 99 %) were purchased from Merck-Sigma Aldrich. All chemicals were used as received without further purification.

Powder XRD, Fourier transform infrared spectroscopy (FTIR), as well as thermogravimetric, and derivative weight loss curves (DTG) data are given for CNP and Tempo treated CNP samples, in Figure S1. The XRD data show a principal diffraction peak positioned at $2\theta = 22^o$, which correspond to the (200) diffraction plane. A single peaks is observed at $2\theta = 16^o$, which is surprising since some literature suggests that two peaks located at $2\theta = 14.9^o$ and $2\theta = 16.7^o$ are normally seen and corresponds to the (1-10) and (110) diffraction planes, respectively. It has been suggested that a single diffraction peak could be explained for crystallite with diamond-shaped cross section [2]. A small diffraction peak was recorded at $2\theta = 35^o$, and corresponds to the (004) diffraction plane. This suggests the cellulose is in its Iβ crystalline form which is typical for plant cellulose [3].

As expected, the FTIR data presented in Figure S1 allowed identifying the presence of carboxylate moieties for CNP TEMPO when compared to CNP due to presence of an absorption peak located at a wavenumber position of 1610 cm^{-1} related to the vibrational motions of carbonyl moieties that belong to the molecular structure of carboxylate. This chemical modification is used to improve the production of individual and monodisperse nanofibers, and results in an increase in the optical transmittance of CNP substrate in the visible region [4].

With respect to TGA traces, one can observe a significant decrease in thermal stability for CNP Tempo compared to CNP. This suggests that CNP and CNP TEMPO substrates have to be exposed to adequate temperature conditions so as to avoid their thermal degradation upon applying further processing or fabrication steps.

Wrinkle Formation

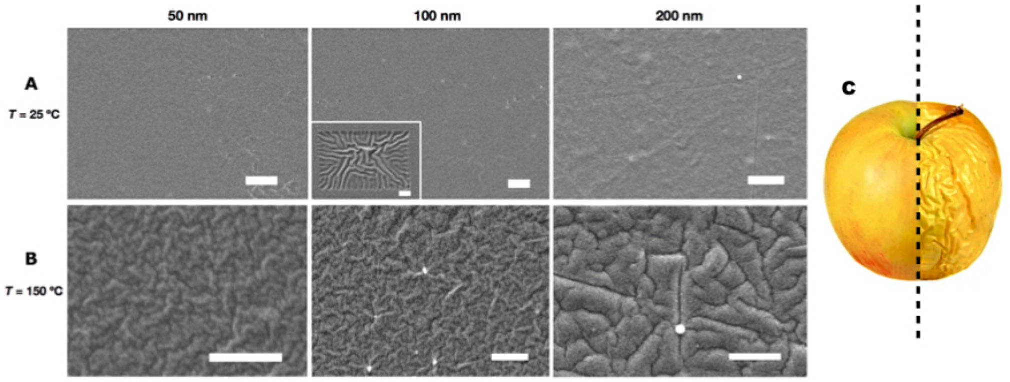

Figure S2. SEM images of TiN coatings on PET substrates produced (A) at room temperature (RT) and T = 150 °C (B). No wrinkles are observed at RT however similar wrinkles can be induced on RT TiN coating by electron-beam irradiation during FE-SEM imaging (inset). Scale bars are 2 µm. (C) picture of a fresh apple (left) and dried apple that exhibit similar skin wrinkles.

The wrinkled-like structures are observed clearly for TiN coatings on PET substrates because of the compressive stress that stems from the important thermal expansion mismatch $\Delta\alpha$ between the thin film and the substrate.This compressive strain $\epsilon = \Delta\alpha\Delta T$ causes the film to wrinkle and then possibly debond from the substrate (for higher deposition

temperature. Examples are given in the Figure S3 where TiN deposited at high temperature produce extreme compressive strain that results in debonding of the coating. When deposited at room temperature, the TiN films are homogeneous and almost no wrinkles are observed as shown in the Figure S2, even for thicker TiN layers.

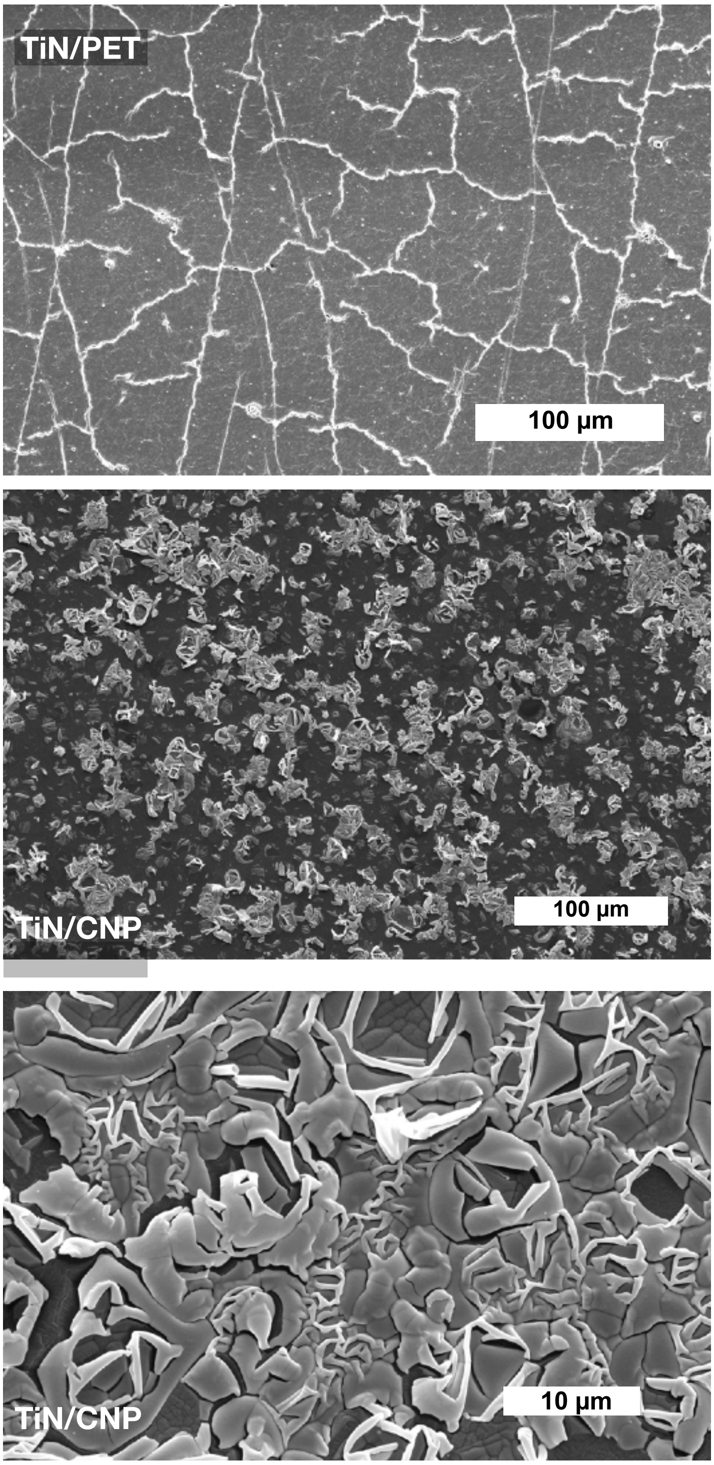

Figure S3. SEM images of TiN coatings on CNP and PET substrate deposited at elevated temperature (T > 200 °C). The thermal degradation of the CNP and PET substrates further increases the compressive strain on the TiN coatings.

References

1. Saito, T.; Isogai, A. TEMPO-Mediated Oxidation of Native Cellulose. The Effect of Oxidation Conditions on Chemical and Crystal Structures of the Water-Insoluble Fractions. *Biomacromolecules* **2004**, *5*, 1983–1989. https://doi.org/10.1021/bm0497769.
2. Duchemin, B. Size, shape, orientation and crystallinity of cellulose I-beta by X-ray powder diffraction using a free spreadsheet program. *Cellulose* **2017**, *24*, 2727–2741. https://doi.org/10.1007/s10570-017-1318-6.
3. French, A.D. Idealized powder diffraction patterns for cellulose polymorphs. *Cellulose* **2014**, *21*, 885–896. https://doi.org/10.1007/s10570-013-0030-4.
4. Lee, K.Y., Ed. *Nanocellulose and Sustainability: Production, Properties, Applications, and Case Studies*; CRC Press, 2018.

Disclaimer/Publisher's Note: The statements, opinions and data contained in all publications are solely those of the individual author(s) and contributor(s) and not of MDPI and/or the editor(s). MDPI and/or the editor(s) disclaim responsibility for any injury to people or property resulting from any ideas, methods, instructions or products referred to in the content.
